# Antimutagenic compounds and their possible mechanisms of action

**DOI:** 10.1007/s13353-014-0198-9

**Published:** 2014-03-11

**Authors:** Karolina Słoczyńska, Beata Powroźnik, Elżbieta Pękala, Anna M. Waszkielewicz

**Affiliations:** 1 Department of Bioorganic Chemistry, Chair of Organic Chemistry, Faculty of Pharmacy, Jagiellonian University Medical College, 9 Medyczna Street, 30-688 Krakow, Poland; 2 Department of Pharmaceutical Biochemistry, Faculty of Pharmacy, Jagiellonian University Medical College, 9 Medyczna Street, 30-688 Krakow, Poland

**Keywords:** Antimutagen, Antimutagenicity, DNA damage, Mutagen, Mutagenicity

## Abstract

Mutagenicity refers to the induction of permanent changes in the DNA sequence of an organism, which may result in a heritable change in the characteristics of living systems. Antimutagenic agents are able to counteract the effects of mutagens. This group of agents includes both natural and synthetic compounds. Based on their mechanism of action among antimutagens, several classes of compounds may be distinguished. These are compounds with antioxidant activity; compounds that inhibit the activation of mutagens; blocking agents; as well as compounds characterized with several modes of action. It was reported previously that several antitumor compounds act through the antimutagenic mechanism. Hence, searching for antimutagenic compounds represents a rapidly expanding field of cancer research. It may be observed that, in recent years, many publications were focused on the screening of both natural and synthetic compounds for their beneficial muta/antimutagenicity profile. Thus, the present review attempts to give a brief outline on substances presenting antimutagenic potency and their possible mechanism of action. Additionally, in the present paper, a screening strategy for mutagenicity testing was presented and the characteristics of the most widely used antimutagenicity assays were described.

## Introduction

The genomes of all living organisms are constantly subjected to damage by both external agents and endogenous processes, such as spontaneous DNA damage. Mutagenicity refers to the induction of permanent changes in the DNA sequence of an organism, which may result in a heritable change in the characteristics of living systems. Mutations may alter a single gene, a block of genes, or whole chromosomes. Point (gene) mutations affect only one nucleotide or a few nucleotides within a gene. Point mutations, which are the most common type of alteration in the DNA sequence, can be divided into three main types: a base pair substitution (the replacement of one base pair with another); a deletion (the loss of one or more base pairs); and an insertion (the addition of extra base pairs into the DNA sequence).

The term “genotoxicity” is a broader concept than mutagenicity and describes the capacity of the compounds to affect the DNA structure or the cellular apparatus and topoisomerases, which are responsible for the genome fidelity. Genotoxic effects on DNA are not always related to mutations (Maurici et al. 2005; Eastmond et al. 2009).

Mutations are created mainly by external factors, including chemical and physical agents, called mutagens. Additionally, mutations can occur spontaneously due to errors in DNA replication, repair, and recombination. In general, mutations can be grouped into negative, neutral, positive, lethal, and sub-lethal. Mutagenic changes that occur in germline cells can be passed to future generations, whereas somatic mutations may contribute to the pathogenesis of various pathological conditions, including cancer (Migliore and Coppedè 2002; Cooke et al. 2003; Izzotti et al. 2003; Weakley et al. 2010).

Antimutagenic agents are able to counteract the effects of mutagens. Therefore, knowledge on the mode of action of certain mutagenic compounds provides a basis for an explanation of how antimutagenic compounds work.

**Fig. 1:**
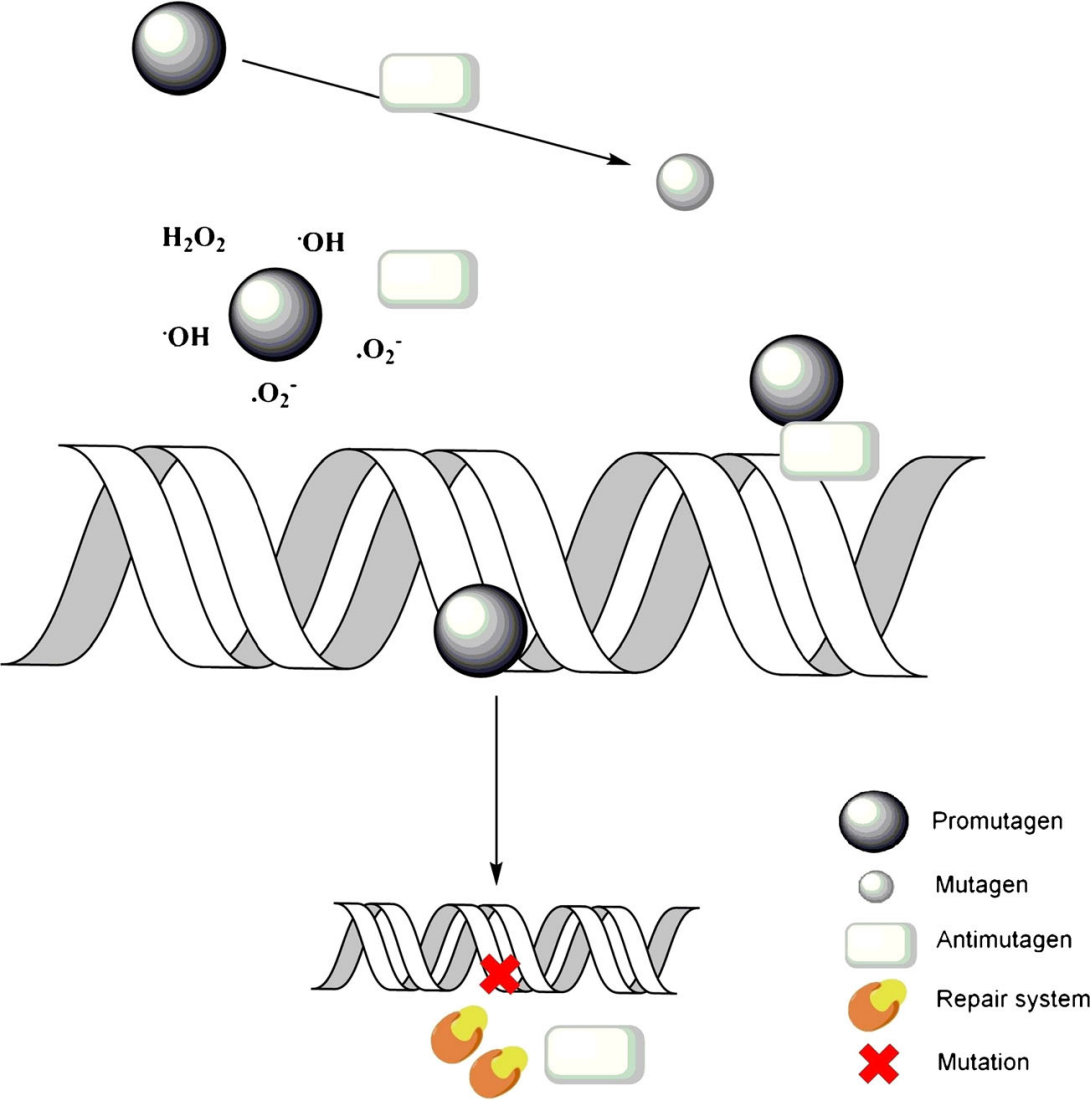
Mechanisms of action of antimutagens

Identifying the antimutagenic compounds is among the most promising area of research in recent years. Therefore, in this review, the substances presenting antimutagenic activity are presented, with special emphasis on their mechanisms of action (Fig. 1). Moreover, the present paper is concerned with the screening strategy for mutagenicity testing and the most popular assays used in antimutagenicity testing.

## Mutagens

The term “mutagen” refers to the chemical or physical agent that is capable of inducing changes in the genetic material of an organism. Consequently, the number of mutation events is increased above the background mutation frequency. As chemical mutagens induce mutations by different mechanisms, several major classes of them, such as alkylating agents, base analogs, and intercalating agents, can be distinguished.

Alkylating agents such as N-methyl-N′-nitro-N-nitrosoguanidine (MNNG) and ethyl methanesulfonate (EMS) are able to react with DNA bases directly and transfer an alkyl group to form monoadducts in genetic material. Consequently, DNA strand breaks are produced, causing specific mispairing (Ralhan and Kaur 2007). The most frequent location of adducts in DNA is at guanine, leading to the formation of O6-alkylguanine (Sanderson and Shield 1996). Noteworthy, some of the alkylating agents, such as cyclophosphamide (CP), are used for the treatment of cancer. Base analogs are molecules that have similar structure to normal DNA bases and, thereby, can substitute a base in genetic material, leading to transitions and tautomerization. For example, 5-bromouracil (5-BU) is an analog of thymine, whereas 2-amino-purine (2-AP) is an analog of adenine. It should be noted that various base analogs are used as anticancer agents and immunosuppressants. Finally, intercalating agents such as acridine mutagen ICR-191 mimic base pairs and are able to insert between DNA bases at the core of the DNA double helix. This results in single-nucleotide pair insertions and deletions.

Many mutagenizing agents known as direct-acting mutagens. such as sodium azide (NaN_3_), affect genetic material directly, leading to structural damage; on the other hand, some compounds, including benzo[α]pyrene (BP), act on DNA in an indirect manner (indirect-acting mutagens) via the induction of the synthesis of different chemicals which influence DNA directly. During this process, the transformation of promutagen into the actual mutagen takes place. Table 1 depicts selected chemical mutagens and their mechanisms of action.

**Table 1:** Selected chemical mutagens and their mechanisms of action

Mutagen	Kind of mutagen	Mechanism of action	Reference
N-acetyl-2-aminofluorene (AAF)	Indirect acting	- Reacts with guanines at the C8 position in DNA to form a structure that interferes with DNA replication	Gill and Romano (2005)
Acridine (AC)	Direct acting	- At low concentrations binds DNA tightly but reversibly by intercalation - At high concentrations induces DNA strand breaks	Ferguson and Denny (2007)
9-aminoacridine (9-AA)	Direct acting	- Induces frameshift mutations at hot spots where a single base, especially guanine, is repeated - Binds to DNA noncovalently by intercalation	Ferguson and Denny (2007) Hoffmann et al. (2003)
2-aminoanthracene (2-AA)	Indirect acting	- Its electrophilic reactive metabolites form DNA adducts	So et al. (2008) Sugamori et al. (2006)
2-aminofluorene (2-AF)	Indirect acting	- Is converted to reactive carcinogenic ester 2-acetylaminofluorene-*N*-sulfate, which can attack guanine residues in nucleic acids	DeBaun et al. (1970)
Aflatoxin B1 (AFB_1_)		- Stimulates the release of free radicals, which cause chromosomal aberrations	Alpsoy et al. (2009)
Benzo(α)pyrene (BP)	Indirect acting	- An active mutagen is benzo[a]pyrene-7,8-diol-9,10-epoxide (BPDE) - Major adducts of BP-DNA are BPDE-deoxyguanosine (dG) and 9-OH-BP-dG-derived adducts	Smith and Gupta (1996)
Cyclophosphamide (CP)	Indirect acting	- Affects DNA through its alkylating properties and free radical production	Zhang et al. (2005)
Doxorubicin (DXN)	Direct acting	- Induces G:C–T:A transversions - Undergoes electron reduction and leads to the generation of free radical species	Koch et al. (1994) Singal et al. (2000)
Ethyl methanesulfonate (EMS)	Direct acting	- An alkylating agent - At low concentrations alters a base in DNA - Induces DNA strand breaks and lesions as a consequence of depurination	Guha and Khuda-Bukhsh (2003) Achary and Panda (2010)
Methyl methanesulfonate (MMS)	Direct acting	- An alkylating agent - Modifies guanine and adenine to cause base mispairing and replication blocks, respectively	Beranek (1990)
N-methyl-N′-nitro-N-nitrosoguanidine (MNNG)	Direct acting	- Leads to the alkylation of purines and pyrimidines - One of the most important products of MNNG is O6-methylguanine	Koch et al. (1994) Kumaresan et al. (1995) Gulluce et al. (2010)
4-nitro-o-phenylenediamine (NPDA)	Direct acting	- Induces frameshift mutations	Koch et al. (1994)
1-nitropyrene (1-NP)	Direct acting	- Forms DNA adduct N-(deoxyguanosine-8-yl)-1-aminopyrene	Bacolod and Basu (2001)
4-nitroquinoline-N-oxide (NQNO)	Direct acting	- A base substitution agent, principally acting at G residues, inducing mainly GC to AT transitions	Fronza et al. (1992)
Sodium azide (NaN_3_)	Direct acting	- Mutagenicity is mediated through the production of an organic metabolite (L-azidoadenine) that enters the nucleus and then interacts with DNA and originates point mutations in the genome - Induces G:C→A:T transitions	Koch et al. (1994) Al-Qurainy and Khan (2009) Gulluce et al. (2010)

## Antimutagens

Certain compounds, known as antimutagens, are able to decrease or even remove the mutagenic effects of potentially harmful chemicals. Novick and Szilard (1952) primarily applied the term “antimutagen” to agents possessing the ability to diminish the rate or frequency of induced or spontaneous mutations. This group of agents includes both natural and synthetic compounds. According to Kada et al. (1982), two different types of antimutagens, i.e., desmutagens and bioantimutagens, can be distinguished. Desmutagens that function extracellularly are able to inactivate mutagenic agents before they reach DNA. On the other hand, bioantimutagens act within the cell and participate in mutation suppression after DNA damage. These compounds are able to influence genome repair and replication (Kada and Shimoi 1987; De Flora 1998). Based on their mechanism of action among antimutagens, several classes of compounds may be distinguished. These are compounds with antioxidant activity; compounds that inhibit the activation of mutagens; blocking agents; as well as compounds characterized with several modes of action. Examples of some recently described antimutagenic compounds and their possible modes of action are presented in Table 2.

**Table 2:** Examples of some recently described antimutagenic compounds and their mechanisms of action

Antimutagen	Mechanism of action	Reference
Cysteine	- Direct chemical interaction with a mutagen	Watanabe et al. (1994)
Gallic acid	- Scavenging of the electrophilic mutagens - Binding or insertion into the outer membrane transporters, leading to the blockage of a mutagen that was transferred into the cytosol	Hour et al. (1999)
Lipoic acid	- Antioxidant potency	Unal et al. (2013)
Phenolics	- Interference with cytochrome P450-mediated metabolism of mutagens - Interaction with active mutagenic metabolites - DNA protection against mutagens presenting electrophilic properties	De Flora et al. (2001) Marnewick et al. (2000)
*Acacia salicina*	- Antioxidant effects - Direct interaction with mutagens electrophilic metabolites - Influence on the enzymes engaged in the metabolism of mutagens	Chatti et al. (2011) Boubaker et al. (2011)
*Acanthopanax divaricatus* var. *albeofructus* (ADA) extracts	- Rapid elimination of mutagenic compounds from the cells before the induction of DNA damage	Hong et al. (2011)
Lichen species	- Antioxidant activity	Nardemir et al. (2013) Agar et al. (2010) Gulluce et al. (2010)
*Mangifera indica* L. stem bark (MSBE)	- Antioxidant activity - Inhibition of the metabolic activation of promutagens	Morffi et al. (2012)
*Phellinus rimosus* extract	- Direct inactivation of mutagens - Inhibition of the metabolic activation of promutagens - Antioxidant potency	Ajith and Janardhanan (2011)
Phytoconstituents from *Terminalia arjuna*	- Inhibition of the metabolic activation of promutagens	Kaur et al. (2010)
Powder of grain (Lisosan G)	- Antioxidant effects	Frassinetti et al. (2012)
Wheat bran	- Antioxidant potency - Modulation of DNA-repairing enzymes	Pesarini et al. (2013)
Xanthones and flavones of *Syngonanthus* (*Eriocaulaceae*)	- Elimination of mutagens from bacteria - Interaction with reactive intermediates of mutagens - The influence on microsomal enzymes	de Oliveira et al. (2013)
β-aminoketones	- Inhibition of the metabolic activation of promutagens - The blockage of mutagens binding to DNA	Gulluce et al. (2010) Hoffmann et al. (2003)
Bichalcophenes	- Binding to DNA and protection against electrophilic mutagens - Interaction with mutagens - Antioxidant activity	Marnewick et al. (2000) Watanabe et al. (1994) Collins et al. (2012)
Luteoline derivatives	- Protection against DNA double-strand breaks - Protection against mutagens intercalating effects or alkylating action	Orhan et al. (2013)
Nitrogen- and oxygen-containing heterocyclic compounds	- Inhibition of the metabolic activation of promutagens	Turhan et al. (2012)
Organoselenium compounds	- Antioxidant potency	Roy et al. (2012)
Pyrrolidine-2,5-dione derivatives	- Direct interaction with a mutagen	Pękala et al. (2013)
Aminoalkanolic derivatives of xanthones	- Direct interaction with a mutagen	Słoczyńska et al. (2010)

It was reported previously that several antitumor compounds act through the antimutagenic mechanism (Tsai et al. 1996; Dion et al. 1997; Ikken et al. 1999). Hence, searching for antimutagenic compounds represents a rapidly expanding field of cancer research (Heo et al. 2001; Ferguson and Philpot 2008; El-Sayed and Hussin 2013; El-Sayed et al. 2013).

Interestingly, certain compounds exhibit dual nature and display both antimutagenic and mutagenic effects. Such compounds are known as “Janus mutagens”, after the Roman god who had one head with two faces looking in opposite directions (von Borstel and Higgins 1998; Zeiger 2003). β-carotene (βCT) belongs to this group of compounds. Its dual nature can be attributed primarily to the fact that βCT possesses the ability to both scavenge and produce free radicals (Paolini et al. 2003).

## Antimutagens with antioxidant potency

As many mutagens act through the generation of reactive oxygen species (ROS), the removal of reactive molecules represents an important strategy in the process of antimutagenesis (Shay et al. 2009; Tian et al. 2012). There is increasing evidence that compounds with antioxidant properties can remove ROS before these molecules react with DNA, resulting in a mutation (Lee et al. 2011; Tian et al. 2012).

Unal et al. (2013), who investigated the antigenotoxic effects of lipoic acid (LA) against mitomycin-C induced chromosomal aberrations, sister chromatid exchanges, and micronucleus formation in human peripheral lymphocytes, demonstrated that LA exhibits both anticlastogenic and antimutagenic activity. The use of several assays in studies on LA antigenotoxicity revealed the comprehensive action of this compound against genetic damage. These beneficial effects can be primarily attributed to the antioxidant potency of LA. Additionally, it was suggested that LA improves the DNA repair system or DNA synthesis. This is consistent with previous reports describing LA as a highly potent antioxidant that plays numerous roles in removing ROS (Evans and Goldfine 2000; Cai et al. 2013; Rochette et al. 2013).

In another study, Nardemir et al. (2013) stated that the antimutagenic action of selected lichen species may be related to the inhibitory activity of the lichen extracts on the formation of free radicals. This was confirmed by its influence on superoxide dismutase (SOD) and glutathione peroxidase (GPx) activity, as well as the glutathione (GSH) and malondialdehyde (MDA) levels. Thus, lichen species may protect DNA from genetic damage through the restoration of natural antioxidant defense mechanisms. Other authors also confirmed that the antimutagenic activity of the lichen extracts is closely related to antioxidant effects (Agar et al. 2010; Kotan et al. 2011). Another example of an antimutagen of natural origin acting mainly through its antioxidant properties is *Acacia salicina*, the extracts of which provide protection against DNA strand scission induced by the hydroxyl radical. The tested extracts decreased significantly the mutagenicity induced by LA and 4-nitro-o-phenylenediamine (NPDA) (Chatti et al. 2011). The observed antigenotoxic potency could be ascribed, at least in part, to their antioxidant effects.

Some antimutagenic compounds are not potent antioxidants on their own but can be converted into molecules that display antioxidant activity. Such phenomena was observed for several amino acid conjugates of curcumin that demonstrated very high antimutagenic activity with mutagens such as NaN_3_ and methyl methanesulfonate (MMS) against *Salmonella typhimurium* strains (Parvathy et al. 2010). Moreover, the antimutagenic activity of a powder of grain (Lisosan G) in yeast *Saccharomyces cerevisiae* was attributed primarily to the antioxidant potency of Lisosan G polyphenols (Frassinetti et al. 2012).

The search for synthetic antimutagens is another important trend in the area of antimutagenicity research. For example, Roy et al. (2012) demonstrated that a series of organoselenium compounds protected against genotoxicity and oxidative stress induced by an indirect-acting mutagen CP (Roy et al. 2012). As CP affects DNA through it alkylating properties and free radicals production (Zhang et al. 2005), the tested compounds may act through multiple antioxidant mechanisms, including the influence on the activity of SOD and catalase (CAT), the level of GSH, and the removal of ROS. Recently, also, the novel bichalcophenes significantly decreased the mutagenicity induced by two mutagens, namely, NaN_3_ and BP (El-Sayed and Hussin 2013). It was found that the antimutagenic potential of the compounds could be attributed to their antioxidant activity (Collins et al. 2012).

Based on current knowledge, antioxidant activity is a desirable property, since it can be attributed to the antimutagenic effects of compounds. Thus, it would be vital to test the antimutagenic potential of any compound that displays antioxidant activity.

## Antimutagens that inhibit the activation of mutagens

The mutagenic effect of promutagens is dependent on their metabolic activation, which is mediated mainly by phase I metabolic enzymes, such as the cytochrome P450 family of enzymes. Some antimutagens are able to inhibit the enzymes responsible for the biotransformation of mutagenic compounds, leading to the inhibition of promutagens bioactivation.

Recently, the antimutagenic potential of some newly synthesized nitrogen- and oxygen-containing heterocyclic compounds against NaN_3_ and MNNG was demonstrated using the Ames/*Salmonella* and *Escherichia coli* WP2 bacterial reverse mutation assay systems (Turhan et al. 2012). The antimutagenic activity of the tested compounds was probably due to the inhibition of L-azidoalanine and O6-methylguanine formation.

With reference to natural antimutagens, Nardemir et al. (2013) observed that the methanol extracts of the lichens have shown antimutagenic effects against NaN_3_, which might result from the extract inhibition of the production of NaN_3_ metabolite, known as L-azidoalanine (Gulluce et al. 2010). In another study, phytoconstituents isolated from *Terminalia arjuna* suppressed the mutagenic effect of the aromatic amine, i.e., 2-aminofluorene (2-AF) (Kaur et al. 2010). The observed activity was found to be a consequence of the inhibition of the metabolic activation of 2-AF to the mutagenic forms. The mutagen activation is connected with N-oxidation by cytochrome P4501A2; next, the activation by N-acetyltransferase takes place (Beudot et al. 1998). Also, in the case of isothiocyanates, the main mechanism of their antimutagenicity is related to the inhibition of the metabolic activation of mutagens via the influence on cytochrome P4501A1 and 1A2 activity (Hamilton and Teel 1995).

## Antimutagens as blocking agents

Another important protective mechanism against chemical mutagenesis is related to the direct chemical interaction between an antimutagenic compound and a mutagen before it induces DNA damage. In that way, 3-chloro-4-(dichloromethyl)-5-hydroxy-2(5H)-furanone (MX) was inactivated using various sulfhydryl compounds, such as cysteine (Watanabe et al. 1994). Blocking agents are also able to prevent mutagenic compounds from reaching target sites. For example, nucleophilic bichalcophenes might be able to bind to DNA and, therefore, protect genetic material from electrophilic mutagenic agents (Marnewick et al. 2000). Another hypothesis for bichalcophenes antimutagenic potential might be that these compounds are able to directly interact with mutagens, leading to the inhibition of their damaging activity (Watanabe et al. 1994).

Hour et al. (1999), who examined the antimutagenic properties of gallic acid by the Ames test, found that this compound could perhaps act as a nucleophile to scavenge the electrophilic mutagens. Moreover, it was implied that gallic acid can bind or insert into the outer membrane transporters and lead to the blockage of a mutagen that was transferred into the cytosol. In another study, *Acanthopanax divaricatus* var. *albeofructus* (ADA) extracts displayed antimutagenic activity against direct-acting mutagenic agents through the rapid elimination of mutagenic compounds from the cells before the induction of genetic material damage (Hong et al. 2011).

## Antimutagens with multiple mechanisms of action

A great variety of antimutagenic agents act through multiple mechanisms to provide protection against diverse mutagens. Noteworthy, the ability of compounds to affect mutagens simultaneously in several different ways significantly increase antimutagenic effectiveness. Hence, searching for such multifunctionally acting antimutagens is of great importance.

In the study conducted by Ozturkcan et al. (2012), the antigenotoxic potential of two newly synthesized β-aminoketones against MNNG and 9-aminoacridine (9-AA)-induced mutagenesis was evaluated. The findings of the study provided information about chemical prevention from the toxicity of both mutagens by using selected compounds. The study elicited that two newly synthesized β-aminoketones, namely, 2-{(4-bromophenyl)[(4-methylphenyl)amino]methyl}cyclohexanone and 2-{(4-chlorophenyl)[(4-methylphenyl)amino]methyl}cyclo-hexanone, demonstrated antimutagenic action against mutagenicity induced by MNNG, a mutagen acting by DNA methylation. The antimutagenic potential of these compounds may be related to the inhibition of the production of O6-methylguanine, a product of MNNG that is related to its mutagenic effect (Eadie et al. 1984; Gulluce et al. 2010). In addition, the study showed that both compounds also abolished mutagenesis induced by 9-AA that binds to DNA noncovalently by intercalation. Consequently, frameshift mutations at hotspots are formed, leading to the repetition of a single base, mainly guanine (Hoffmann et al. 2003). Thus, the antimutagenic effect of β-aminoketones might be explained on the basis of the blockage of mutagen binding to DNA.

In another study, Ajith and Janardhanan (2011) demonstrated the in vitro antimutagenic activity of ethyl acetate extract of macro fungus, *Phellinus rimosus*, using the Ames assay. It was concluded that the antimutagenic potential of the extract against direct-acting mutagens may result from the direct inactivation of mutagens. It is probable that, due to stimulation of the transmembrane export system in bacteria, mutagenic compounds are removed from the cells before they influence the DNA structure. Additionally, in the case of doxorubicin (DXN), the extract of *P. rimosus* may affect the intercalation of mutagens to genetic material. The antimutagenic effect of the extract against indirect-acting mutagen BP may be partially ascribed to the inhibition of the mixed-function oxidase (MFO) system and also to the conjugation of the components of the extract with benzo[a]pyrene-7,8-diol-9,10-epoxide (BPDE), being a BP-active mutagen. Moreover, the inhibition of 2-AF-induced mutagenesis might be related to the MFO inhibition or inactivation of the reactive carcinogenic ester of 2-AF, namely, 2-acetylaminofluorene-N-sulfate, which is capable of attacking guanine residues in nucleic acids. In case of both types of mutagens (direct and indirect), the extract of *P. rimosus* may remove free radical species generated by certain mutagens, such as DXN and BP.

Boubaker et al. (2011) demonstrated that extracts of *Acacia salicina* display potent antioxidant and antimutagenic activities. Chloroform extract was antimutagenic against both direct- and indirect-acting mutagens, as the extract may serve as a blocking agent that is capable of influencing the activities of enzymes engaged in the metabolism of mutagens and carcinogens. Moreover, the tested extract displayed the ability to react directly with the mutagen’s electrophilic metabolites and was capable of protecting against oxidative DNA damage.

In another study, Morffi et al. (2012) investigated the antimutagenic effects of *Mangifera indica* L. stem bark (MSBE) extract against DNA damage induced by ten different mutagenic agents in the Ames test. MSBE is a Cuban nutraceutical supplement rich in polyphenols. It was observed that MSBE protected against genetic material damage induced by all the tested mutagens, except for NaN_3_. This DNA protection may be due to the antioxidant activity of MSBE. In addition, the antimutagenic properties of the extract may be explained by the influence of MSBE upon the CYP subfamily. Pesarini et al. (2013) examined the antimutagenic effects of wheat bran and concluded that such properties may be mainly related to the presence of the antioxidant phytic acid. It was demonstrated that phytic acid may intercept carcinogenic azoxymethane, inhibiting it even before it can damage DNA. Moreover, antioxidants included in wheat bran are able to modulate DNA repair enzymes.

In the case of heterocyclic aromatic amines (HAAs), it was proved that the attenuation of their unfavorable mutagenic effect might result from the influence on the DNA repair pathway, the stimulation of detoxifying enzymes, and the inhibition of enzymes that participate in the metabolic activation of HAAs (Schwab et al. 2000).

Phenolics are able to act against mutagens via both intracellular and extracellular mechanisms (De Flora 1998; De Flora et al. 2001). The extracellular mechanism involves interference with the cytochrome P450-mediated metabolism of these mutagens and the interaction with active mutagenic metabolites (Marnewick et al. 2000). Furthermore, the antimutagenic potency of these compounds may be related to DNA protection from mutagens presenting electrophilic properties (Marnewick et al. 2000).

In another experiment, the antimutagenic potential of luteoline derivatives (luteolin-7-O-glucoside, luteolin-7-O-rutinoside, and luteolin-7-O-glucuronide) against acridine (AC) was explained by the fact that these derivatives are able to stop the production of DNA double-strand breaks or AC intercalating effects. In addition, the inhibition effects against ethyl methanesulfonate (EMS) may be related to the protection against DNA double-strand breaks or EMS alkylating action (Orhan et al. 2013).

The antimutagenic potential of xanthones and flavones of *Syngonanthus* (*Eriocaulaceae*) was stated with recombinant yeast assay (RYA) and the Ames test (de Oliveira et al. 2013). This beneficial activity may be attributed to different mechanisms, such as the rapid elimination of mutagens from bacteria; the interaction between antimutagens and the reactive intermediates of mutagens; and the influence on microsomal enzymes.

With reference to synthetic compounds, in our team, we evaluated the antimutagenic activity of some aminoalkanolic derivatives of xanthones and some new derivatives of pyrrolidine-2,5-dione with antiepileptic activity (Słoczyńska et al. 2010; Pękala et al. 2013). These compounds were tested with the *Vibrio harveyi* assay against direct mutagen 4-nitroquinoline-N-oxide (NQNO). According to the results obtained, two of the tested xanthone derivatives presented beneficial antimutagenic potential. As for derivatives of pyrrolidine-2,5-dione, some of them had strong or moderate antimutagenic activity against NQNO. In general, one may speculate that the core structures of the test compounds may suggest their possible interactions with NQNO, thus preventing mutagenic activity, similarly to previously reported mechanisms of antimutagenic activities of caffeine and other methylxanthines (Ulanowska et al. 2005, 2007; Ulanowska and Węgrzyn 2006).

In summary, it seems that the interest in antimutagenic substances displaying multiple mechanisms of action is determined by the universality of their action and will be an important trend in the research and development of new antimutagenic compounds in the near future.

## Mutagenicity testing strategy

For any compound that is a candidate for use as a therapeutic agent, it is vital that it does not display mutagenic potency. Additionally, compounds presenting antimutagenic properties may be able to modulate or reduce the mutagenic effects of some chemicals.

In the field of drug discovery, mutagenicity data are required for the pharmaceuticals before the commencement of clinical trials and marketing authorization. The screening strategy for mutagenicity testing is based on a battery of tests and includes both in vitro and in vivo assays, according to the results obtained. The above approach ensures that a wide variety of genetic damage such as gene mutation, chromosomal damage, and aneuploidy can be identified. Noteworthy, both in vitro and in vivo testing methods are used to identify the same endpoints. The European Union has already implemented this strategy; additionally, guidelines have been recommended internationally (Combes et al. 2007).

In general, mutagenicity assessment can be divided into three phases. Phase 1 is based upon in vitro tests that are performed with cultured bacterial and mammalian cells; Phase 2 involves the assessment of mutagenic activity in vivo in somatic cells; and, finally, Phase 3 assays screen for germ cell mutagens (Eastmond et al. 2009; Valdiglesias et al. 2010). Recommended protocols for the suitable tests are given in the Organisation for Economic Co-operation and Development (OECD) guidelines and the International Workshops on Genotoxicity Testing (IWGT) guidance.

Phase 1 assays employ bacteria and mammalian cells and are used for the identification of gene mutations and chromosome alterations. In the early mutagenicity assessment, two or three different tests in bacteria and mammalian cells should be used. The bacterial mutation assays such as *Salmonella typhimurium* and *Escherichia coli* WP2 reverse mutation tests are a useful tool for point mutations identification. These assays allow for the detection of new mutations which are able to revert old mutations existing in tester strains.

Mammalian mutation assays are useful especially in case of bactericidal compounds and agents acting preferentially on the replication system in mammals. Common Phase 1 in vitro mammalian tests include: the mouse lymphoma thymidine kinase (TK) gene mutation assay, which detects compounds that induce forward gene mutations in the *tk* gene of the L5178Y mouse lymphoma cell line, and the hypoxanthine guanine phosphorybosyl transferase (HPRT) gene mutation assay, which identifies agents that cause gene mutations in the *hprt* gene of a suitable cell line, such as Chinese hamster cells (Combes et al. 2007; Eastmond et al. 2009; Johnson 2012).

With reference to chromosomal abnormalities detection, both structural and numerical changes can be identified in vitro in metaphase-spread preparations from exposed mammalian cells. Common in vitro chromosomal damage tests include the mammalian chromosome aberration test and the micronucleus test. In the former assay, mammalian metaphase cells are analyzed for the presence of structural chromosome aberrations, and in the latter, micronuclei in the cytoplasm of cultured mammalian cells during interphase is detected. The micronucleus test is a procedure for the detection of both aneuploidy and clastogenicity in cultured mammalian cells (Combes et al. 2007; Eastmond et al. 2009).

Phase 2 in vivo assays can be used in the verification of the positive results obtained in Phase 1 testing. The common procedure is searching for cytogenetic damage with the use of metaphase analysis assay or the micronucleus test. The in vivo chromosome aberration test in mammals allows the identification of structural chromosome changes induced by a substance in the bone marrow cells of animals, whereas the in vivo micronucleus assay is used for the identification of genetic changes induced by the tested compound to the chromosomes or the mitotic apparatus of cells by the analysis of erythrocytes as sampled in the bone marrow and/or peripheral blood cells of animals. Other in vivo assays include transgenic animal assays for point mutations, which can be used for the simultaneous detection of mutagenic effects in various tissues; DNA strand breakage assays, such as a comet assay (also referred to as the single-cell gel electrophoresis assay), which detect single- and double-strand breaks, repair induced breaks and alkali-labile lesions; and the liver unscheduled DNA synthesis (UDS) test, which is useful for the measurement of the repair of DNA lesions (Combes et al. 2007; Eastmond et al. 2009).

Compounds that give positive results for mutagenic potential in somatic cells in vivo should be further tested with germ cells. Germ cell assays available in Phase 3 fall into two classes. Class 1 includes assays in germ cells per se, such as gene mutation tests in transgenic animals; paternal germinal mutation in the expanded simple tandem repeat (ESTR) test; and chromosomal aberration tests. On the other hand, class 2 contains assays used for the identification of alterations in the offspring of exposed animals. These studies include i.a. testing for gene mutations in th ESTR assay; mouse visible specific locus test for detecting and quantifying the induction of heritable point mutations (intragenic changes and small deficiencies) in mammals; the biochemical specific locus test which allows the detection of mutations originating in the germ line of a mammalian species; and for chromosome or gene mutations in the dominant lethal test (Verhofstad et al. 2008; Eastmond et al. 2009). Table 3 depicts the characteristics of the most popular bioassays used to assess the mutagenicity of compounds.

**Table 3:** Characteristics of the most popular bioassays used to assess the mutagenicity of compounds

Phase	Test name	Endpoint	Reference
1	*Salmonella typhimurium* reverse mutation test	Gene mutations in bacteria	OECD (1997a) Test Guideline 471
1	*Escherichia coli* WP2 reverse mutation test	Gene mutations in bacteria	OECD (1997a) Test Guideline 471
1	In vitro mouse lymphoma test	Gene mutations in mammalian cells	OECD (1997e) Test Guideline 476
1	Hypoxanthine guanine phosphorybosyl transferase (HPRT) gene mutation assay	Gene mutations in mammalian cells	OECD (1997e) Test Guideline 476
1	In vitro mammalian cell micronucleus test	Structural and numerical chromosome alterations	OECD (2010) Test Guideline 487
1	In vitro mammalian chromosome aberration test	Chromosome aberrations	OECD (1997b) Test Guideline 473
1	In vitro comet assay	DNA damage	Burlinson (2012)
1	*Saccharomyces cerevisiae* gene mutation assay	Gene mutations in yeast	OECD (1986a) Test Guideline 480
2	Mammalian erythrocyte micronucleus test	Structural and numerical chromosome alterations	OECD (1997c) Test Guideline 474
2	Mammalian bone marrow chromosome aberration test	Structural chromosome aberrations	OECD (1997d) Test Guideline 475
2	Transgenic animal assays for point mutations	Gene mutations	IWGT Test Guideline
2	In vivo comet assay	DNA damage	Burlinson et al. (2007) Burlinson (2012)
2	Unscheduled DNA synthesis (UDS) test with mammalian liver cells in vivo	DNA damage	OECD (1997g) Test Guideline 486
3	Transgenic animal assays for point mutations	Gene mutations	IWGT Test Guideline
3	DNA mutation in expanded simple tandem repeat (ESTR) test		Singer et al. (2006)
3	Mammalian spermatogonial chromosome aberration test	Structural chromosome aberrations	OECD (1997f) Test Guideline 483
3	Mouse visible specific locus test	Gene mutations	Russell et al. (1981)
3	Mouse biochemical specific locus (MBSL) test	Gene mutations	Lewis et al. (1986)
3	Rodent dominant lethal test	Gene mutations and chromosome changes	OECD (1984) Test Guideline 478
3	Mouse heritable translocation assay	Structural and numerical chromosome changes	OECD (1986b)Test Guideline 485

## Antimutagenicity screening assays

Usually, the antimutagenicity assay is done as the appropriate mutagenicity test, except that the tested cells are treated simultaneously with both the test compound and a standard mutagen. In the early evaluation of the antimutagenic effects of compounds, basic bacterial short-term assays are used. These assays have many advantages, including their simplicity, relatively low cost, sensitivity, and flexibility to different experimental settings (De Flora et al. 1992). In addition, such tests enable to indicate the possible mechanisms of antimutagenic activity. Listed below are only the tests that are most frequently used to screen compounds for antimutagenic activity.

The Ames test, also known as the *Salmonella typhimurium*/microsome assay (Maron and Ames 1983), is one of the most widely used short-term mutagenicity/antimutagenicity test. The assay detects the mutagenic potential of tested substances through the induction of reverse mutations in the *his* operon of genetically modified *S. typhimurium* strains (Maron and Ames 1983; Mortelmans and Zeiger 2000). The test detects mutagenic agents acting with different mutation mechanisms, such as base-pair substitution and frameshift mutations. Moreover, by using tester strains with different genotypes, the antimutagenic activity of compounds against mutations induced by various mutagenic agents that act via different mechanisms can be evaluated (Mortelmans and Zeiger 2000). *Salmonella typhimurium* mutagenicity and antimutagenicity test procedures can all be applicable to the *Escherichia coli* WP2 reverse mutation assay. The only assay difference is the addition of trace amounts of tryptophan instead of histidine to the top agar. This assay is primarily useful in the detection of A/T base pair damage (Mortelmans and Riccio 2000).

In the last several decades, several rapid bacterial mutagenicity/antimutagenicity tests have been developed and optimized, such as the assay based on a marine bacterium *Vibrio harveyi* (Czyż et al. 2000, 2002; Piosik et al. 2003; Węgrzyn and Czyż 2003; Podgórska et al. 2005; Ulanowska and Węgrzyn 2006; Słoczyńska et al. 2010; Kamiński et al. 2013; Pękala et al. 2013). The test employs a series of genetically modified *Vibrio harveyi* strains. The bacterium is naturally sensitive to neomycin; however, antibiotic-resistant mutants can be separated. The frequency of appearance of mutants increases in the presence of mutagens in a dose–response manner, and this forms the basis of this assay.

Another vital tool in antimutagenicity assessment is the SOS chromotest (Quillardet and Hofnung 1985). As with the other above-mentioned tests, this test was also developed as an alternative to the Ames test. The SOS chromotest is a colorimetric assay that employs *Escherichia coli* PQ37 mutant strain and allows the assessment of DNA changes induced by various mutagens by the measurement of the expression of a reporter gene, β-galactosidase (Quillardet et al. 1985).

Finally, the antimutagenicity assay on yeasts is also very popular in searching for new antimutagens. This is mainly due to the fact that yeasts as eukaryotes are characterized with chromosome structure and DNA repair processes similar to those in mammals. Furthermore, *Saccharomyces cerevisiae* strains are equipped with endogenous cytochrome P450, and, therefore, can be very useful when testing promutagens (Zimmermann et al. 1975). Table 4 provides an overview of the main advantages and disadvantages of the most popular tests used in preliminary antimutagenicity assessment.

**Table 4:** Comparison of the advantages and disadvantages of the most widely used antimutagenicity screening tests

Test name	Main advantages	Main disadvantages
*Salmonella typhimurium* assay	- Very extensive database available - Easy to perform - No special equipment is necessary	- Tester organism is a potentially pathogenic bacterium - Several tester strains should be used - A relatively long time necessary to perform the analysis - Will not detect mutagens that interact with eukaryote-specific targets
*Escherichia coli* WP2 assay	- Easy to perform - No special equipment is necessary - Only one tester strain is needed	- A relatively long time necessary to perform the analysis - Will not detect mutagens that interact with eukaryote-specific targets
*Vibrio harveyi* assay	- Relatively low cost - The simplicity of procedures - Tester organism is not pathogenic to humans - May detect significantly lower concentrations of typical chemical mutagens than the Ames test - No special equipment is necessary	- Several tester strains should be used - A relatively long time necessary to perform the analysis - Will not detect mutagens that interact with eukaryote-specific targets
SOS chromotest	- The simplicity of procedures - Test rapidity - Only one tester strain is needed	- Will not detect mutagens that interact with eukaryote-specific targets - Special equipment is necessary
*Saccharomyces cerevisiae* assay	- Eukaryotic architecture - *Saccharomyces cerevisiae* strains do have endogenous cytochrome P450 - No special equipment is necessary	- A relatively long time necessary to perform the analysis

## Conclusions

Mutagenic activity is one of the most important endpoints for the risk assessment of chemical compounds, including drug substances and drug candidates, as mutagens are capable of inducing various kinds of changes in the genetic material of a cell. On the other hand, the mutagenic effects of some chemicals may be partly modulated or reduced by the use of compounds presenting antimutagenic properties.

Research over the past few years has revealed that mutation has a key role in carcinogenesis. Therefore, one may expect that searching for compounds with antimutagenic potency will remain in the focus of research in the near future. Research studies on antimutagenicity should be focused primarily on the understanding of the mode of action of the most active compounds. Furthermore, there is still much more research needed in order to clear up the exact links between the results of the short-term antimutagenicity studies and anticarcinogenicity experiments in animal models.
